# Some Information Geometric Aspects of Cyber Security by Face Recognition

**DOI:** 10.3390/e23070878

**Published:** 2021-07-09

**Authors:** C. T. J. Dodson, John Soldera, Jacob Scharcanski

**Affiliations:** 1 School of Mathematics, University of Manchester, Manchester M13 9PL, UK; 2 Federal Institute of Education, Science and Technology Farroupilha, Santo Ângelo 98806-700, Brazil; John.Soldera@iffarroupilha.edu.br; 3 Institute of Informatics, Federal University of Rio Grande do Sul, Porto Alegre 91501-970, Brazil; jacobs@inf.ufrgs.br

**Keywords:** entropy, information geometry, cyber security, classification, feature recognition, retrieval, 53B20, 62M40, 60D05

## Abstract

Secure user access to devices and datasets is widely enabled by fingerprint or face recognition. Organization of the necessarily large secure digital object datasets, with objects having content that may consist of images, text, video or audio, involves efficient classification and feature retrieval processing. This usually will require multidimensional methods applicable to data that is represented through a family of probability distributions. Then information geometry is an appropriate context in which to provide for such analytic work, whether with maximum likelihood fitted distributions or empirical frequency distributions. The important provision is of a natural geometric measure structure on families of probability distributions by representing them as Riemannian manifolds. Then the distributions are points lying in this geometrical manifold, different features can be identified and dissimilarities computed, so that neighbourhoods of objects nearby a given example object can be constructed. This can reveal clustering and projections onto smaller eigen-subspaces which can make comparisons easier to interpret. Geodesic distances can be used as a natural dissimilarity metric applied over data described by probability distributions. Exploring this property, we propose a new face recognition method which scores dissimilarities between face images by multiplying geodesic distance approximations between 3-variate RGB Gaussians representative of colour face images, and also obtaining joint probabilities. The experimental results show that this new method is more successful in recognition rates than published comparative state-of-the-art methods.

## 1. Introduction

It is probable that the widest use of cyber security software is in face and fingerprint recognition, with perhaps a billion or more users of phones, tablets and laptops thereby gaining daily access to their devices. The classification and searching of digital datasets for retrieving images or other objects usually will require multidimensional methods because the features used in classification depend on statistically distributed data. Information geometry provides a natural Riemannian metric structure on smooth spaces of probability density functions. This means that changing properties of a dataset or a subset thereof can be represented on a trajectory in the space of distributions with a natural distance function monitoring the changes. Very high dimensional datasets can be projected onto smaller spaces of features by dimensionality reduction, via eigenvalues of the positive definite symmetric matrices of inter-feature distances [1].

In the context of data represented via probability distributions, multivariate Gaussian distributions are a common choice in representing features in complex large datasets, in consequence of their maximal entropy for given mean and covariance; we outline their geometry in Section 2. We described in [2,3] an efficient method for colour face image recognition using information geometry in such a way that each face image was represented by a set of 3-variate Gaussians, one for the vicinity of each landmark point in the face. The three variables are the RGB colours of pixels and we used sums of geodesic distance approximations between them at corresponding landmarks of distinct images to measure dissimilarities between face images. Such geodesic distance approximations between *k*-variate Gaussians are presented in Section 3. Here in Section 4 we describe a new face recognition method which represents face dissimilarities via the product of such geodesic distances and via joint probabilities. This new method proves to be better than comparable state-of-the-art other face recognition methods.

## 2. Multivariate Gaussian Distributions

In the classification of large sets of digital data objects, a common practical choice is the numerical representation of individual features through multivariate Gaussian distributions, which have a maximal entropy property among distributions with a given mean vector and covariance matrix. Then we have, as described below, an information metric on the space of such multivariate Gaussian probability density functions and we can retrieve all objects with a given feature near to that of a chosen object.

The *k*-variate Gaussian distributions have the parameter space

$$R^{k}⊕R^{(k^{2}+k)/2}$$

with probability density functions f(x;μ,Σ) given by:

(1) $$f\left(x;\mu ,\Sigma \right)=\frac{e^{-\frac{1}{2}{\left(x-\mu \right)}^{T}\Sigma^{-1}\left(x-\mu \right)}}{\sqrt{{\left(2\pi \right)}^{k}\left|\Sigma \right|}},$$

where $x\in R^{k}$ is a possible value for the random variable, $\mu \in R^{k}$ a *k*-dimensional mean vector, and $\Sigma \in R^{(k^{2}+k)/2}$ is the k×k positive definite symmetric covariance matrix, for features with *k*-dimensional representation [4]. In such cases the parameters are obtained using maximum likelihood estimation, as was the case for face recognition applications [3]. The Riemannian manifold of the family of *k*-variate Gaussians for a given *k* is well understood through information geometric study using the Fisher information metric. For an introduction to information geometry and a range of applications see [5,6,7].

The Fisher information metric is a Riemannian metric defined on a smooth statistical manifold whose points are probability measures from a probability density function. The Fisher metric determines the geodesic distance between between points in this Riemannian manifold. Given a statistical manifold with coordinates $\theta =(\theta_{1},\theta_{2},\ldots ,\theta_{n})$, and a probability density function p(x,θ) as a function of θ, the Fisher information metric is defined as:

(2) $$g_{jk}\left(\theta \right)=\int_{X}\frac{\partial \log p(x,\theta )}{\partial \theta_{j}}\frac{\partial \log p(x,\theta )}{\partial \theta_{k}}p\left(x,\theta \right)\;dx,$$

which can be understood as the infinitesimal form of the relative entropy and it is also related to the Kullback-Leibler divergence [6,7]. Moreover, a closed-form solution for the Fisher information distance for *k*-variate Gaussian distributions is still unknown [8]. The entropy of the *k*-variate Gaussian (1) is maximal for a given covariance Σ, and mean μ, and it is independent of translations of the mean

(3) $$H\left(\mu ,\Sigma \right)=-\int_{0}^{\infty }f\left(x\right)\log f\left(x\right)\;dx=\frac{1}{2}\log{\left(2\pi e|\Sigma |\right)}^{k}.$$

The natural norm on mean vectors is

(4) $$\left||\mu |\right|=\sqrt{\mu^{T}\Sigma^{-1}\mu }$$

and the eigenvalues ${\left\{\lambda_{i}\right\}}_{i=1\ldots k}$ of Σ yield a norm on covariances:

(5) $$\left||\Sigma |\right|=\sqrt{\sum_{i}^{k}{\left(\lambda_{i}\right)}^{2}}$$

The information distance, that is the length of a geodesic, between two *k*-variate Gaussians $f^{A}$ and $f^{B}$ is the infimum over the length of curves from $f^{A}$ to $f^{B}.$ It is known analytically in three particular cases:

**Diagonal covariance matrix:**$\Sigma =Diag(\sigma_{1},\ldots ,\sigma_{k}):$$f^{A}=\left(k,\mu^{A},\Sigma^{A}\right),f^{B}=\left(k,\mu^{B},\Sigma^{B}\right)$

Here Σ is a diagonal covariance matrix with null covariances [8]:

(6) $$D_{\sigma }\left(f^{A},f^{B}\right)=\sqrt{2\sum_{i=1}^{k}{\left(\log\;s\frac{\left|\left(\frac{{\mu_{i}}^{A}}{\sqrt{2}},{\sigma_{i}}^{A}\right)-\left(\frac{{\mu_{i}}^{B}}{\sqrt{2}},-{\sigma_{i}}^{B}\right)\right|+\left|\left(\frac{\mu_{i}^{A}}{\sqrt{2}},{\sigma_{i}}^{A}\right)-\left(\frac{\mu_{i}^{B}}{\sqrt{2}},{\sigma_{i}}^{B}\right)\right|}{\left|\left(\frac{{\mu_{i}}^{A}}{\sqrt{2}},{\sigma_{i}}^{A}\right)-\left(\frac{{\mu_{i}}^{B}}{\sqrt{2}},-{\sigma_{i}}^{B}\right)\right|-\left|\left(\frac{{\mu_{i}}^{A}}{\sqrt{2}},{\sigma_{i}}^{A}\right)-\left(\frac{{\mu_{i}}^{B}}{\sqrt{2}},{\sigma_{i}}^{B}\right)\right|}\right)}^{2}}.$$

**Common covariance matrix:** $\Sigma^{A}=\Sigma^{B}=\Sigma :$ $f^{A}=\left(k,\mu^{A},\Sigma \right),f^{B}=\left(k,\mu^{B},\Sigma \right)$

Here Σ is a positive definite symmetric quadratic form and gives a norm on the difference vector of means:

(7) $$D_{\mu }\left(f^{A},f^{B}\right)=\sqrt{{\left(\mu^{A}-\mu^{B}\right)}^{T}\cdot \Sigma^{-1}\cdot \left(\mu^{A}-\mu^{B}\right)}.$$

**Common mean vector:** $\mu^{A}=\mu^{B}=\mu :$ $f^{A}=\left(k,\mu ,\Sigma^{A}\right),f^{B}=\left(k,\mu ,\Sigma^{B}\right)$

In this case we need a positive definite symmetric matrix constructed from $\Sigma^{A}$ and $\Sigma^{B}$ to give a norm on the space of differences between covariances. The appropriate information metric is given by Atkinson and Mitchell [9] from a result attributed to S.T. Jensen, using

$$S^{AB}={\Sigma^{A}}^{-1/2}\cdot \Sigma^{B}\cdot {\Sigma^{A}}^{-1/2},\;\;\mathrm{with}\;\;\left\{\lambda_{j}^{AB}\right\}=\mathrm{Eig}\left(S^{AB}\right)\;\;\mathrm{so}$$

(8) $$\begin{array}{ccc} D_{\Sigma }\left(f^{A},f^{B}\right) & = & \sqrt{\frac{1}{2}\sum_{j=1}^{k}\log^{2}\left(\lambda_{j}^{AB}\right)}. \end{array}$$

In principle, (8) yields all of the geodesic distances since the information metric is invariant under affine transformations of the mean [9] Appendix 1; see also the article of P. S. Eriksen [10].

In cases where we have only empirical frequency distributions, and empirical estimates of moments, we can use the Kullback-Leibler divergence, also called relative entropy, between two *k*-variate distributions

$$f^{A}=\left(x;\mu^{A},\Sigma^{A}\right),f^{B}=\left(x;\mu^{B},\Sigma^{B}\right)$$

with given mean and covariance matrices, its square root yields a separation measurement [11,12]:

(9) $$\begin{array}{ccc} KL(f^{A},f^{B}) & = & \frac{1}{2}\log\left(\frac{\det\Sigma^{B}}{\det\Sigma^{A}}\right)+\frac{1}{2}\mathrm{Tr}\left[\Sigma^{B^{-1}}\cdot \Sigma^{A}\right]\\ & + & \frac{1}{2}{\left(\mu^{A}-\mu^{B}\right)}^{T}\cdot \Sigma^{B^{-1}}\cdot \left(\mu^{A}-\mu^{B}\right)-\frac{k}{2}. \end{array}$$

This is not symmetric, so to obtain a distance we can take the average KL-distance in both directions:

(10) $$D_{KL}\left(f^{A},f^{B}\right)=\sqrt{\frac{|KL\left(f^{A},f^{B}\right)|+|KL\left(f^{B},f^{A}\right)|}{2}}$$

The Kullback-Leibler distance tends to the Fisher information distance as two distributions become closer together; conversely it becomes less accurate as they move apart. Using only the first and last term in (11) together with (10), we define a divergence $DKL_{\Sigma }\left(f^{A},f^{B}\right)$ by

(11) $$\begin{array}{ccc} DKL_{\Sigma }\left(f^{A},f^{B}\right) & = & \frac{1}{2}\left(\sqrt{\left|\frac{1}{2}\log\left(\frac{\det\Sigma^{B}}{\det\Sigma^{A}}\right)+\frac{1}{2}\mathrm{Tr}\left[\Sigma^{-B}.\Sigma^{A}\right]-\frac{k}{2}\right|}\right.\\ & & +\left.\sqrt{\left|\frac{1}{2}\log\left(\frac{\det\Sigma^{A}}{\det\Sigma^{B}}\right)+\frac{1}{2}\mathrm{Tr}\left[\Sigma^{-A}.\Sigma^{B}\right]-\frac{k}{2}\right|}\right). \end{array}$$

The Kullback-Leibler divergence does in fact induce the Fisher metric [5,6]. However, there are other geometries with known closed-form solutions for the geodesic distance between *k*-variate Gaussians such as the one defined by the $L^{2}$-Wasserstein metric which is derived by the optimal transport problem in which the mass of one distribution is moved to the other [13]. In this geometry, the space of Gaussian measures on a Euclidean space is geodesically convex and corresponds to a finite dimensional manifold since Gaussian measures are parameterized by means and covariance matrices. By restricting it to the space of Gaussian measures inside the $L^{2}$-Wasserstein space, giving a Riemannian manifold which is geodesically convex, several authors derived a closed-form solution for the distance between two such Gaussian measures A,B, for example Takatsu [13]:

(12) $$W{\left(f_{A},f_{B}\right)}^{2}=\left|\mu_{A}-\mu_{B}\right|+Tr\left[\Sigma^{A}\right]+Tr\left[\Sigma^{B}\right]-2Tr{\left[{\Sigma^{A}}^{\frac{1}{2}}\Sigma^{B}{\Sigma^{A}}^{\frac{1}{2}}\right]}^{\frac{1}{2}}.$$

Additionally, Bhatia et al. [14] used the Bures-Wasserstein distance on the space of *k*-variate Gaussian distributions with zero means in the form:

(13) $$BW{\left(f_{A},f_{B}\right)}^{2}=Tr\left[\Sigma^{A}\right]+Tr\left[\Sigma^{B}\right]-2Tr{\left[{\Sigma^{A}}^{\frac{1}{2}}\Sigma^{B}{\Sigma^{A}}^{\frac{1}{2}}\right]}^{\frac{1}{2}}.$$

## 3. Geodesic Separation between *k*-Variate Gaussians

Using the results in Section 2 from [15], we investigated in [2,3] the following possible choices for approximating the geodesic distance between two *k*-variate Gaussians $F_{1},F_{2}$ with arbitrary means:

(14) $$\begin{array}{ccc} G_{\mu }^{g}\left(F_{1},F_{2}\right) & = & 0.5\sqrt{{\left(\mu_{1}-\mu_{2}\right)}^{T}{\left(\Sigma_{1}\right)}^{-1}\left(\mu_{1}-\mu_{2}\right)}\\ & + & 0.5\sqrt{{\left(\mu_{1}-\mu_{2}\right)}^{T}{\left(\Sigma_{2}\right)}^{-1}\left(\mu_{1}-\mu_{2}\right)}, \end{array}$$

and,

(15) $$G_{\mu }^{h}\left(F_{1},F_{2}\right)=\sqrt{{\left(\mu_{1}-\mu_{2}\right)}^{T}{\left(\frac{\Sigma_{1}+\Sigma_{2}}{2}\right)}^{-1}\left(\mu_{1}-\mu_{2}\right)}\;.$$

From (8), $G_{\Sigma }\left(F_{1},F_{2}\right)$ between the covariances at fixed mean is given by:

(16) $$\begin{array}{ccc} G_{\Sigma }\left(F_{1},F_{2}\right) & = & \sqrt{\frac{1}{2}\sum_{j=1}^{k}\log^{2}\left(\lambda_{j}^{12}\right)}\;\;\mathrm{with}\;\;S^{12}={\Sigma_{1}}^{-1/2}\cdot \Sigma_{2}\cdot {\Sigma_{1}}^{-1/2},\\ & & \;\mathrm{and}\;\left\{\lambda_{j}^{12}\right\}=\mathrm{Eig}\left(S^{12}\right). \end{array}$$

This led to two distinct ways to approximate the geodesic distance between *k*-variate Gaussians,

(17) $$\begin{array}{ccc} G_{g}\left(F_{1},F_{2}\right) & = & \frac{G_{\mu }^{g}\left(F_{1},F_{2}\right)+G_{\Sigma }\left(F_{1},F_{2}\right)}{2},\;\;\mathrm{or} \end{array}$$

(18) $$\begin{array}{ccc} G_{h}\left(F_{1},F_{2}\right) & = & \frac{G_{\mu }^{h}\left(F_{1},F_{2}\right)+G_{\Sigma }\left(F_{1},F_{2}\right)}{2}. \end{array}$$

In the context of face recognition, dissimilarity metrics can be very useful to measure dissimilarities between face images or between patches of face images. Accordingly, geodesic distance approximations such as $G_{g}$ and $G_{h}$, Equations (17) and (18), can be employed as a dissimilarity metric between probability distributions representative of face landmarks [2,3] as we show in the face recognition approach that we present next.

## 4. Face Recognition Experiments

The distance between two Gaussian distributions lying in the Riemannian manifold of *k*-variate Gaussians is given by the arc length of a minimizing geodesic curve which connects both Gaussians. Moreover, geodesics are intrinsic geometric objects and they are invariant under smooth transformations of coordinates, so in particular the length of a segment is invariant under scale changes of the random variables, from which the mean vectors and covariances are computed.

Consequently, geodesic distances play the role of a natural dissimilarity metric in biometric applications which represent features by probability distributions such as face recognition [2,3]. In such applications, landmark topologies can be used to locate and extract compact biometric features from characteristic face locations in high resolution colour face images [16,17].

Since an analytic form for the geodesic distance in the Riemannian manifold of *k*-variate Gaussians is currently unknown, here we approximate it by constructing approximations applied in a set of face recognition experiments with features represented as *k*-variate Gaussians.

In order to extract efficient features for face recognition, we used the FEI Face database [18], which provides colour (RGB) face images with 640×480 pixels. The database images were taken against a white homogenous background, with the head in the upright position, turning from left to right, and there are varying illumination conditions and face expressions. Since the images are 3-channeled (RGB), so here k=3.

Also, we made use of another challenging database, namely the FERET Face Database [19], which provides colour (RGB) face images with 512×768 pixels organized in several subsets with specific head pose, expression, age, and illumination conditions.

**Figure 1:**
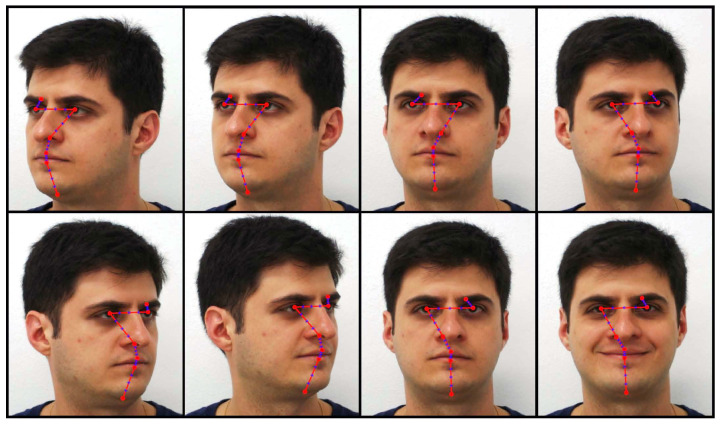
Adopted landmark topology in the FEI Face Database with varying face poses and expressions [18].

To extract meaningful features from face images of both databases, we adopted the landmark topology presented in Figure 1 with seven landmarks at characteristic face locations such as eyebrows, eyes, nose, mouth and chin (in red dots), together with three equally spaced interpolated landmarks between each pair of consecutive landmarks (in blue), leading to a total of L=25 landmarks for each face image. Next, all pixels inside squared patches with size 11×11 centred at each landmark location are extracted, leading to a feature space dimensionality of 3025 pixels.

However, it is possible to reduce this high-dimensionality feature space and preserve its discriminative properties by representing each landmark *ℓ* by the 3-dimensional mean $\mu_{\ell }$ and the 3-variate covariance matrix $\Sigma_{\ell }$ of each extracted face patch, using images with three colour channels (RGB). Accordingly, each landmark is represented by 9 dimensions (3 from the mean and 6 from the covariance matrix since it is symmetric). As result, the original feature space dimensionality is reduced to 225. Experimentally, the optimally small landmark topology, interpolated landmark number *L* and vicinity size were determined, leading to the landmark number L=25 and square patches with size 11×11 pixels.

Therefore, by representing each face image as an ordered sequence of probability distributions as in previous approaches [2,3], dissimilarities between distinct face images were scored by summing geodesic distances between 3-variate Gaussians representative of corresponding landmarks of pairs of face images *x* and *y*. Differently here, we obtained an improved score function for dissimilarities between face images by multiplying the geodesics between corresponding landmarks as follows. We define the functions:

(19) $$\mathrm{Using}\;\left(\mu ,Diag\left(\Sigma \right)\right):\;\;\;{S_{d}}^{x,y}=\prod_{l=1}^{L}D_{\sigma }\left(F_{x}^{\ell },F_{y}^{\ell }\right),$$

(20) $$\mathrm{Using}\;\left(\mu ,\Sigma \right):\;\;\;{S_{g}}^{x,y}=\prod_{l=1}^{L}G_{g}\left(F_{x}^{\ell },F_{y}^{\ell }\right),$$

(21) $$\mathrm{Using}\;\left(\mu ,\Sigma \right):\;\;\;{S_{h}}^{x,y}=\prod_{l=1}^{L}G_{h}\left(F_{x}^{\ell },F_{y}^{\ell }\right),$$

(22) $$\mathrm{Using}\;\left(\mu ,\Sigma \right):\;\;\;{S_{w}}^{x,y}=\prod_{l=1}^{L}W\left(F_{x}^{\ell },F_{y}^{\ell }\right).$$

where $F_{x}^{\ell }$ and $F_{y}^{\ell }$ represent 3-variate Gaussians, $F_{x}^{\ell }\left(\mu_{x}^{\ell },\Sigma_{x}^{\ell }\right)$ and $F_{y}^{\ell }\left(\mu_{y}^{\ell },\Sigma_{y}^{\ell }\right)$, respectively, *ℓ* is the $\ell^{th}$ landmark from a total of *L* landmarks, and ${S_{d}}^{x,y}$, ${S_{g}}^{x,y}$, ${S_{h}}^{x,y}$, ${S_{w}}^{x,y}$ are score functions applicable to images *x* and *y*. Clearly, in our experiments we cannot use the Bures-Wasserstein distance, Equation (13), since we measure varying means for our RGB variables, but the Wasserstein distance, Equation (12), is suitable and we tested it with the score in Equation (22). Equation (12) might be worth investigating further in future work, as might be a hybrid distance, $G_{BW}+D_{\mu }$ using Equations (7) and (13).

All the aforementioned scores define face dissimilarities as products of individual landmark dissimilarities given by geodesic distances. However, by considering a face matching problem, it is possible to convert such dissimilarities between landmarks into probabilities of landmarks not matching, as follows:

(23) $$P{\left(x,y\right)}^{\ell }=\frac{G(F_{x}^{\ell },F_{y}^{\ell })}{\sum_{m=1}^{M}G\left(F_{x}^{\ell },F_{m}^{\ell }\right)},$$

where *m* represents the *m*-th candidate face image from a total of *M* available face images, and *G* is a chosen dissimilarity metric. Then, the problem of finding the face image $F_{y}$ which is more similar to $F_{x}$ is converted into the problem of finding the face image $F_{y}$ which has the least probability of not matching $F_{x}$. This probability is defined as the joint probability of not matching for all landmarks, i.e., the product of the probabilities of not matching each landmark *ℓ* as follows:

(24) $$\mathrm{Using}\;\left(\mu ,Diag\left(\Sigma \right)\right):\;\;\;P_{d}\left(x,y\right)=\prod_{l=1}^{L}\frac{D_{\sigma }\left(F_{x}^{\ell },F_{y}^{\ell }\right)}{\sum_{m=1}^{M}D_{\sigma }\left(F_{x}^{\ell },F_{m}^{\ell }\right)},$$

(25) $$\mathrm{Using}\;\left(\mu ,\Sigma \right):\;\;\;P_{g}\left(x,y\right)=\prod_{l=1}^{L}\frac{G_{g}\left(F_{x}^{\ell },F_{y}^{\ell }\right)}{\sum_{m=1}^{M}G_{g}\left(F_{x}^{\ell },F_{m}^{\ell }\right)},$$

(26) $$\mathrm{Using}\;\left(\mu ,\Sigma \right):\;\;\;P_{h}\left(x,y\right)=\prod_{l=1}^{L}\frac{G_{h}\left(F_{x}^{\ell },F_{y}^{\ell }\right)}{\sum_{m=1}^{M}G_{h}\left(F_{x}^{\ell },F_{m}^{\ell }\right)},$$

(27) $$\mathrm{Using}\;\left(\mu ,\Sigma \right):\;\;\;P_{w}\left(x,y\right)=\prod_{l=1}^{L}\frac{W(F_{x}^{\ell },F_{y}^{\ell })}{\sum_{m=1}^{M}W\left(F_{x}^{\ell },F_{m}^{\ell }\right)}.$$

We can also provide an informal interpretation of our three methods: joint probabilities, sums or products of geodesic distances over the set of L=25 landmarks. By defining the problem of matching one face to another in terms of corresponding landmark dissimilarities, such dissimilarities are converted into probabilities of landmarks not matching as previously presented. Then, by multiplying individual probabilities of landmarks not matching, we obtain the joint probability of all landmarks not matching together at the same time. However, the sum of such probabilities of distinct sequenced events does not have much statistical meaning in our case. Accordingly, by multiplying the landmark dissimilarities, the impact of very similar landmarks is greatly increased as well as very dissimilar landmarks, and the same occurs in the joint probability which also multiplies such landmark dissimilarities. Finally, the product of geodesics has a formulation very similar to the joint probability up to a normalizing factor unique for each test face image.

Finally, the classification procedure is according to the nearest neighbour rule, which means that a new test face sample is attributed to the database individual which presents the training sample that minimizes the chosen score function $S_{d}$, $S_{g}$, $S_{h}$, $S_{w}$, or joint probability $P_{d}$, $P_{g}$, $P_{h}$, $P_{w}$. Even with large datasets, this classification rule has presented low computational complexity due to the fact that we calculate geodesic distance approximations between *k*-variate Gaussians, with a small *k* value, i.e., k=3, allowing the proposed method to operate near real time as further detailed [2].

In order to validate these new score functions and our geodesic product distance approximations, face recognition experiments were performed to compare our methods with state-of-the-art comparative methods. In the experiments with the FEI face database [18], the first 100 individuals were selected considering the eight head poses indicated in Figure 1, which include the frontal neutral and smiling expressions. Ten runs were performed with the selected database images, and in each run, seven head poses per individual were randomly selected for training, and the remaining one was selected for testing. The averaged recognition rates for the proposed method and comparative methods are presented in Table 1, with all methods using features extracted from the landmark topology shown in Figure 1.

Additionally, an extended set of experiments was performed in the FERET face database [19] by using the first 150 individuals which present the subsets fa, fb, hl, hr, ql and qr, which are like the head poses and face expressions in Figure 1. Ten runs were performed with the selected database images, and in each run, five head poses per individual were randomly selected for training, and the remaining one was selected for testing. The averaged recognition rates for the proposed method and comparative methods are also presented in Table 1, with all methods using features extracted from the landmark topology shown in Figure 1. Some of the comparative methods presented in this Table have parameters, so the parameter values which maximized their recognition rates were experimentally determined to obtain their final recognition rates. Those methods are outlined briefly below.

The Eigenfaces method [20] linearly approximates the inherently non-linear face manifold by creating a orthogonal linear projection which best preserves the global feature geometry. On other hand, the Fisherfaces method [21] determines a linear projection which maximizes the between class covariance while minimizing the within class covariance, leading to a better class separation. Furthermore, the method Customized Orthogonal Laplacianfaces (COLPP) [17] obtains an orthogonal linear projection onto a discriminative linear space, which better preserves both the data and class geometry.

In another linear approach, the Multi-view Discriminant Analysis (MvDA) method [22] seeks for a single discriminant common space for multiple views in a non-pairwise manner by jointly learning multiple view-specific linear transforms. In the CCA method [23], multiple feature vectors are fused to produce a feature vector that is more robust to the weakness of each individual vector. And the Coupled Discriminant Multi-manifold Analysis (CDMMA) method [24] explores the neighbourhood information as well as the local geometric structure of the multi-manifold space.

Although the linear approach is simple and efficient, it is also possible to approximate the non-linear face manifold by using non-linear approaches like the Enhanced ASM method [16], which estimates the most discriminative landmarks and scores face similarities by summing probabilities associated to each landmark, taking advantage of this natural multi-modal feature representation. It turned out that the geodesic sum method [2,3] improves on this approach by more accurately scoring face dissimilarities by summing geodesic distances between corresponding landmarks of distinct face images. The experimental results presented in Table 1 include our new methods, geodesic products using the score functions $S_{g}$, $S_{h}$, $S_{d}$, $S_{w}$, and joint probabilities using $P_{g}$, $P_{h}$, $P_{d}$, $P_{w}$, which use our geodesic distance approximations between landmarks on face images. Finally, we performed experiments with the method CM (Continuous Model) [25], summing dissimilarities from corresponding landmarks by using Mahalanobis distance.

**Table 1:** Averaged recognition rates of comparative face recognition methods in the FEI Face database [18] and the FERET Face database [19] using colour (RGB) face images and the landmark topology presented in Figure 1.

Method	FEI	FERET
Joint Probabilities (with $P_{g}$) Equation (17)	99.50%	97.13%
Joint Probabilities (with $P_{h}$) Equation (18)	99.50%	96.86%
Joint Probabilities (with $P_{d}$) Equation (6)	90.00%	79.26%
Joint Probabilities (with $P_{w}$) Equation (12)	96.70%	87.06%
Geodesic Products (with $S_{g}$) Equation (17)	99.50%	97.13%
Geodesic Products (with $S_{h}$) Equation (18)	99.50%	96.86%
Geodesic Products (with $S_{d}$) Equation (6)	90.00%	79.26%
Geodesic Products (with $S_{w}$) Equation (12)	96.70%	87.06%
Geodesic Sums [3], uses Equation (17)	99.50%	96.80%
Geodesic Sums [3], uses Equation (18)	99.50%	96.73%
Geodesic Sums [3], uses Equation (6)	88.40%	75.93%
Geodesic Sums [3], uses Equation (12)	87.30%	81.40%
CM [25]	98.60%	92.33%
Enhanced ASM [16]	89.20%	69.20%
CCA [23]	70.90%	29.06%
CDMMA [24]	37.70%	12.26%
MvDA [22]	44.40%	20.13%
COLPP [17]	96.10%	88.66%
LDA [21]	87.20%	66.00%
Eigenfaces [20]	82.20%	52.00%

## 5. Conclusions

From the experiments reported in Table 1, the geodesic product distance approximation $S_{g}$ Equation (17) for 3-variate Gaussians provided the best recognition rate in all experiments, overcoming comparative state-of-the-art methods and also confirming its efficiency as a dissimilarity metric applied in face recognition.

Another conclusion based on Table 1 is that recognition rates with the geodesic distance approximations $S_{g}$ and $S_{h}$ are better than with $S_{d}$ (and $P_{g}$ and $P_{h}$ are better than with $P_{d}$) mainly because they take account of local covariances among RGB values in the face images while $S_{d}$ and $P_{d}$ ignore all covariances, leading to the conclusion that covariances increase the reliability of geodesic distance approximations between 3-variate Gaussians.

Moreover, scores $S_{g}$ and $S_{h}$ and joint probabilities $P_{g}$ and $P_{h}$ based on our geodesic distance approximations applied in face recognition also achieved higher recognition rates than scores $S_{w}$ and joint probabilities $P_{w}$ based on the Wasserstein metric, helping to confirm the efficiency of the Fisher metric [6] over other geometries for such distributions in our case, since the Fisher metric better accounts the geometry of the *k*-variate Gaussian distributions because this metric measures the amount of information variation of probability distributions in relation to its parameters, in our case, individual means and covariances.

Finally, the results show that the product of geodesic distances (and joint probabilities) can more accurately score dissimilarities between 3-variate face feature representations than just summing such dissimilarities, since by multiplying landmark dissimilarities the impact of very similar landmarks is greatly increased as well as very dissimilar landmarks, increasing the reliability of face recognition.

## Data Availability

Not Applicable.
